# An Ultra-Sensitive Electrochemical Sensor for the Detection of Carcinogen Oxidative Stress 4-Nitroquinoline N-Oxide in Biologic Matrices Based on Hierarchical Spinel Structured NiCo_2_O_4_ and NiCo_2_S_4_; A Comparative Study

**DOI:** 10.3390/ijms21093273

**Published:** 2020-05-05

**Authors:** Tse-Wei Chen, Elayappan Tamilalagan, Shen-Ming Chen, Muthumariappan Akilarasan, Selvarasu Maheshwaran, Xiaoheng Liu

**Affiliations:** 1 Department of Materials, Imperial College London, London SW7 2AZ, UK; coolman91339@gmail.com; 2 Department of Chemical Engineering and Biotechnology, National Taipei University of Technology, Taipei 106, Taiwan; tamilalagane2014@gmail.com (E.T.); m.akil.arasann@gmail.com (M.A.); maheshwarans333@gmail.com (S.M.); 3 Key Laboratory of Education Ministry for Soft Chemistry and Functional Materials, Nanjing University of Science and Technology, Nanjing 210094, China

**Keywords:** 4-NQO detection, NiCo_2_O_4_, NiCo_2_S_4_, biologic samples, hierarchical spinel structure, hydrothermal method

## Abstract

Various factors leads to cancer; among them oxidative damage is believed to play an important role. Moreover, it is important to identify a method to detect the oxidative damage. Recently, electrochemical sensors have been considered as the one of the most important techniques to detect DNA damage, owing to its rapid detection. However, electrode materials play an important role in the properties of electrochemical sensor. Currently, researchers have aimed to develop novel electrode materials for low-level detection of biomarkers. Herein, we report the facile hydrothermal synthesis of NiCo_2_O_4_ micro flowers (MFs) and NiCo_2_S_4_ micro spheres (Ms) and evaluate their electrochemical properties for the detection of carcinogen-causing biomarker 4-nitroquinoline n-oxide (4-NQO) in human blood serum and saliva samples. Moreover, as-prepared composites were fabricated on a glass carbon electrode (GCE), and its electrochemical activities for the determination of 4-NQO were investigated by using various electrochemical techniques. Fascinatingly, the NiCo_2_S_4_-Ms showed a very low detection limit of 2.29 nM and a wider range of 0.005 to 596.64 µM for detecting 4-NQO. Finally, the practical applicability of NiCo_2_S_4_-Ms in the 4-NQO spiked human blood serum and saliva samples were also investigated.

## 1. Introduction

Currently, bimetallic oxide and sulfide-based nanoparticles have been widely used in the field of electrochemical application [1,2,3]. A well-known electrode material with superior electrocatalytic property prepared based on nickel and cobalt due its higher surface area, structures and unique morphologies than monometallic oxides or sulfides [4,5,6,7,8]. Thus, we have synthesized hierarchical spinel NiCo_2_O_4_-MFs and NiCo_2_S_4_-Ms as an electrode material for the electrocatalytic reduction of 4-NQO detection. The better response occurs on the NiCo_2_S_4_-Ms modified electrode because sulfides provide the lower bandgap energy and less electronegativity than oxides [8,9,10]. The synthesis method for NiCo2S4 and NiCo2O4 with various structures such as, micro flowers, nanoprism [11], nanoplates [12] and nanorods [13] and their electrocatalytic properties were previously reported. However, the hydrothermal method are more convenient to synthesis nanomaterials with different morphology then other available methods [14,15,16]. The chemical reaction in hydrothermal process occurs due to the influential high-temperature and pressure, which results the formation of exceptional products [17].

The term oxidative stress is defined to be the imbalance or the excessive production of reactive oxygen species (ROS) in cellular metabolism [18,19]. It plays an important role in living organisms such as maintenance of cell growth and production of important biologic substances, etc. [20]. However, in living organisms free radicals such as nitric oxide, peroxynitrite produce the ROS, which undergoes nitration in protein molecules, forms the 4-nitroquinoline n-oxide [21]. Thus, the excess level of 4-NQO leads to various types of disorders such as cancer, neurodegeneration and lung diseases, etc. [22]. Therefore, it is essential and important to develop analytical techniques to identify the oxidative stress biomarker 4-NQO in biologic matrices. The current analytical techniques that are used to determine 4-NQO ions are fluorescence-based screening assay, high performance liquid chromatography (HPLC) and electrochemical methods [22]. Among the aforementioned techniques, electrochemical techniques are easy to handle, require low consumption of samples, are rapid and most importantly, are low-cost [22,23]. Moreover, the fabrication of the electrode surface with the suitable materials will precisely improve the electrochemical parameters such as stability and sensitivity [24].

Herein, we reported that the facile synthesis of hierarchical spinel structure of NiCo_2_S_4_ microspheres and NiCo_2_O_4_ micro flowers for the effective detection of oxidative stress biomarker 4-NQO. The as-prepared NiCo_2_S_4_-Ms shows the excellent electrochemical activities for the determination of 4-NQO on comparing with NiCo_2_O_4_-MFs. Furthermore, the differential pulse voltammetry (DPV) results exhibited a low limit of detection and a wider linear range for the detection of biomarker 4-NQO. Moreover, the prepared NiCo_2_S_4_-Ms shows excellent long-term stability and selectivity. Finally, real samples of the sensor were tested in 4-NQO spiked human blood serum and saliva samples.

## 2. Results and Discussion

### 2.1. Microscopic and Elemental Analysis of Synthesized Nanomaterials

**Figure 1:**
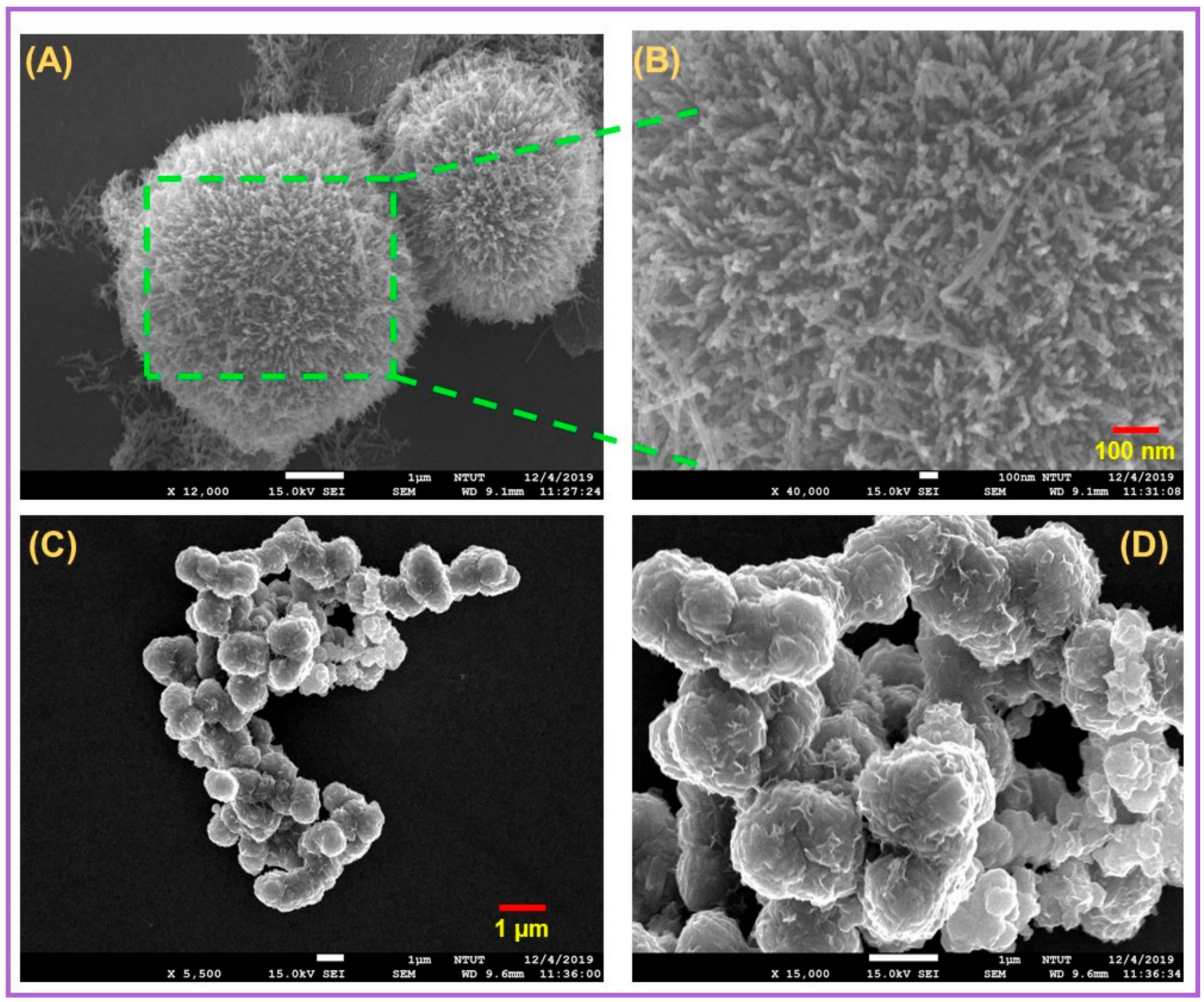
(**A**) FE-SEM of NiCo_2_O_4_-MFs, (**B**) magnified image of NiCo_2_O_4_-MFs and (**C**,**D**) NiCo_2_S_4_-Ms. Scale bar 100 nm.

**Figure 2:**
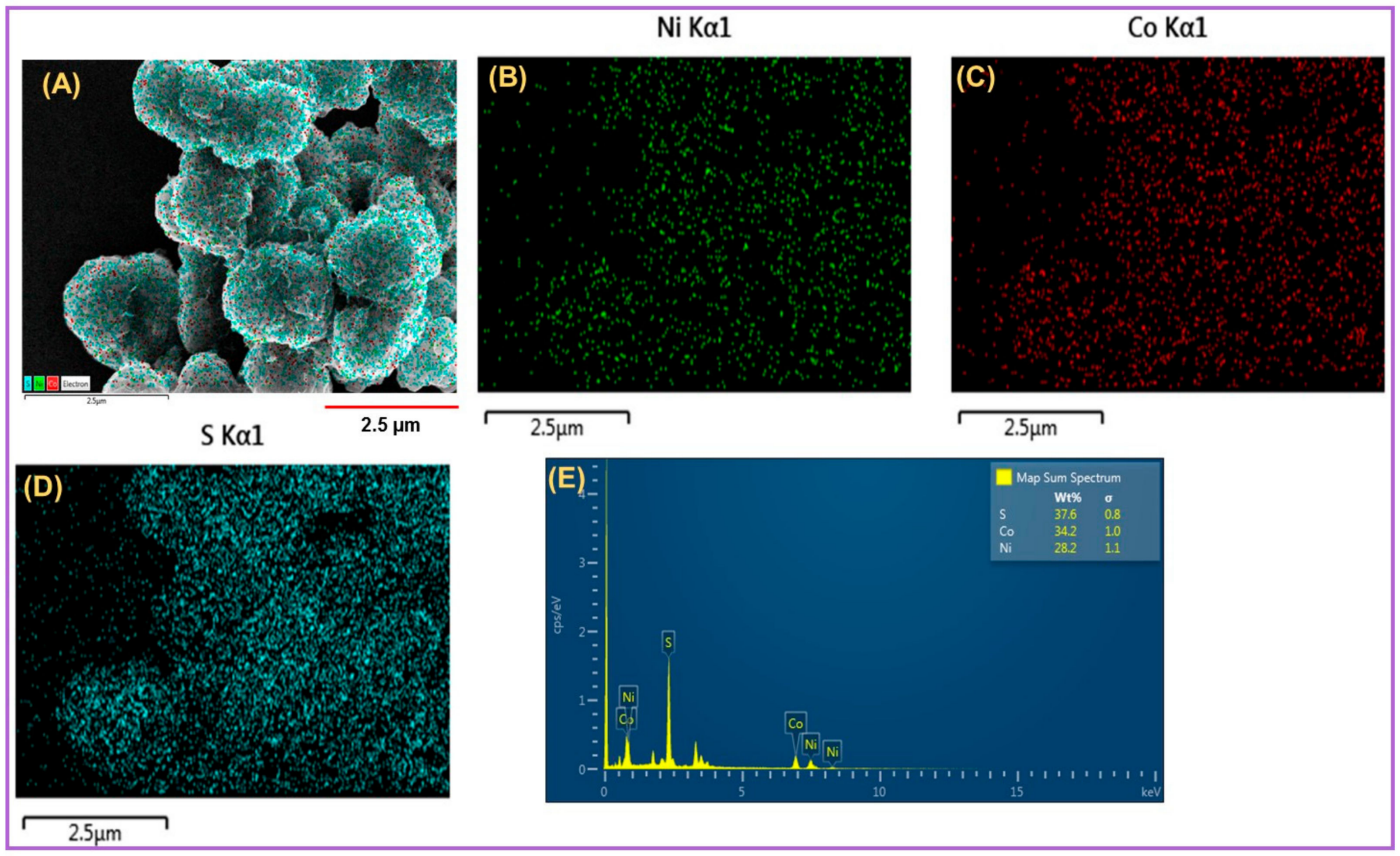
(**A**) Mapping image NiCo_2_S_4_-Ms mix, (**B**) Ni, (**C**) Co and (**D**) S. (**E**) EDX profile of NiCo_2_S_4_-Ms. Inset weight percentage of NiCo_2_S_4_-Ms. Scale bar 2.5 μm.

The morphologic structure of synthesis nanoparticles micro flowers and microspheres were obtained using FESEM. Figure 1A characteristic the morphology of NiCo_2_O_4_, which appears as a flower-like structure and Figure 1B, shows the magnified microscopic image of NiCo_2_O_4_ in which clearly demonstrated that the numerous nano-needles are shrinks together and forms a micro spiky structure. Moreover, the FESEM images of the NiCo_2_S_4_ Figure 1C,D show that obtained particles were micro structured spheres, which were regularly distributed with an average particle size of 1.64 µm. Figure 2A–D illustrates the elemental mapping of NiCo_2_S_4_ Ms (A), which confirmed the presence of Ni (B), Co (C) and S (D) elements. In addition, the EDX analysis were taken to investigate the elemental composition present in NiCo_2_S_4_ Ms. Figure 2E shows the EDX spectrum of NiCo_2_S_4_ Ms with the expected signal response of the elements Ni, Co and S. The inset image of Figure 2E presents the weight percent NiCo_2_S_4_ Ms. Moreover, the elemental mapping and EDX spectrum including the weight percentage of NiCo_2_O_4_ MFs are presented in supplementary Figure S1A–E.

### 2.2. XRD and XPS Analysis of NiCo_2_S_4_-Ms and NiCo_2_O_4_-MFs

**Figure 3:**
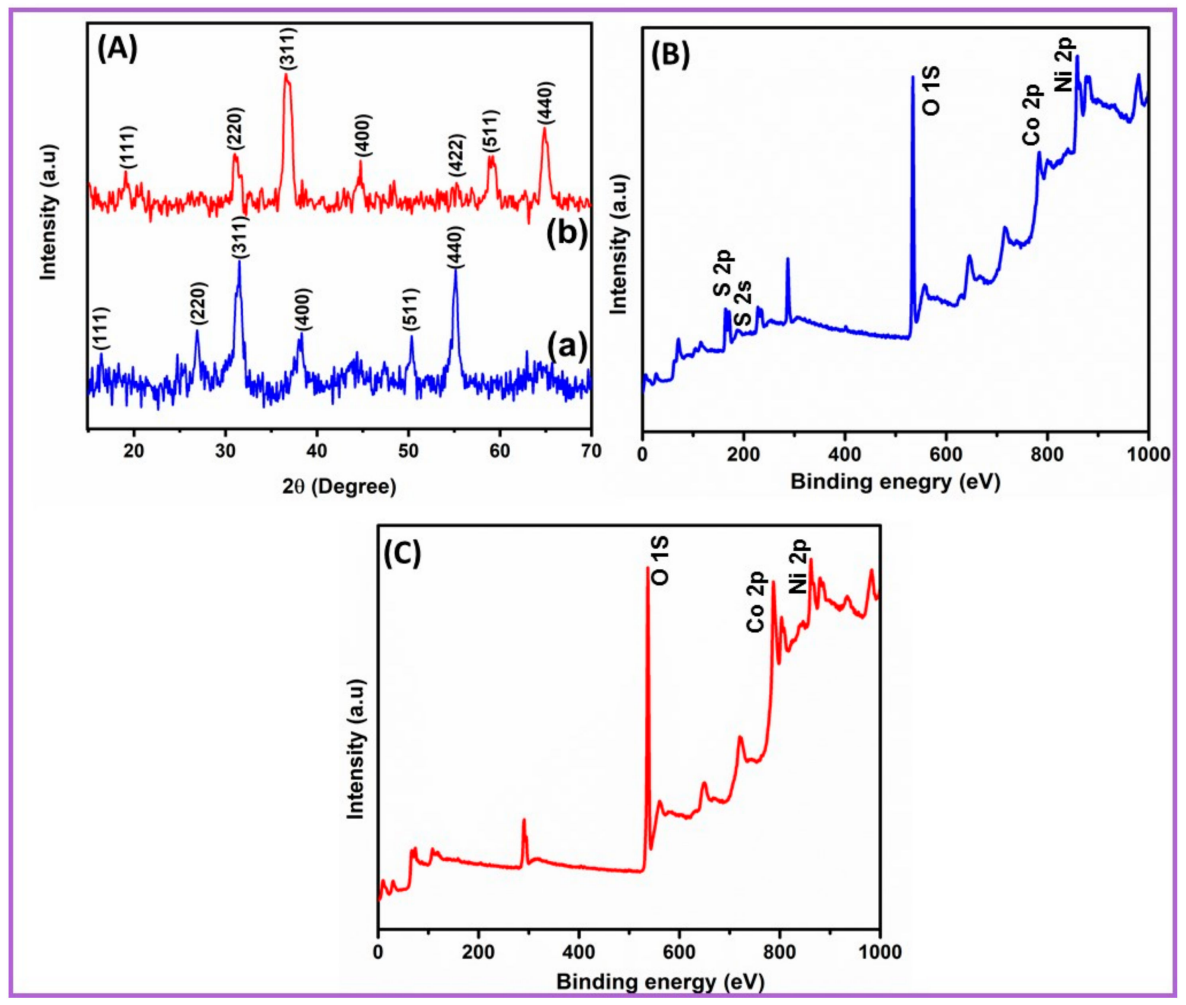
(**A**)—(**a**) XRD study of NiCo_2_S_4_-Ms and (**b**) NiCo_2_O_4_-MFs. (**B**) XPS survey spectrum of NiCo_2_S_4_-Ms and (**C**) NiCo_2_O_4_-MFs.

The XRD patterns of the as synthesized NiCo_2_S_4_-Ms and NiCo_2_O_4_-MFs are shown in Figure 3A. The XRD pattern of NiCo_2_S_4_-Ms Figure 3Aa shows the characteristic peaks at 16.3^°^ (111), 26.8^°^ (220), 31.6^°^ (311), 38.3^°^ (400), 50.4^°^ (511), 55.3^°^ (440). These peaks were reliable with the standard XRD pattern of NiCo_2_S_4_-Ms (JCPDS NO: 20-0782) [25]. Moreover, Figure 3Ab shows the signal response at 18.9^°^ (111), 31.1^°^ (220), 36.7^°^ (311), 44.6^°^ (400), 55.4^°^ (422), 59.1^°^ (511), 64.9^°^ (440) shows the maximum crystallization, which confirms the formation of NiCo_2_O_4_-MFs (JCPDS NO: 20-0781) [26]. In addition, the XPS were taken to conform the chemical composition and the oxidation state of NiCo_2_S_4_-Ms and NiCo_2_O_4_-MFs. Figure 3B,C shows the XPS survey spectra of NiCo_2_S_4_-Ms and NiCo_2_O_4_-MFs confirms that, the presence of Ni, Co, S and O [27,28]. Thus, the XRD and XPS studies clearly confirms that the excellent formation of NiCo_2_S_4_-Ms and NiCo_2_O_4_-MFs.

### 2.3. EIS and Electrochemical Investigation of Different Electrodes

**Figure 4:**
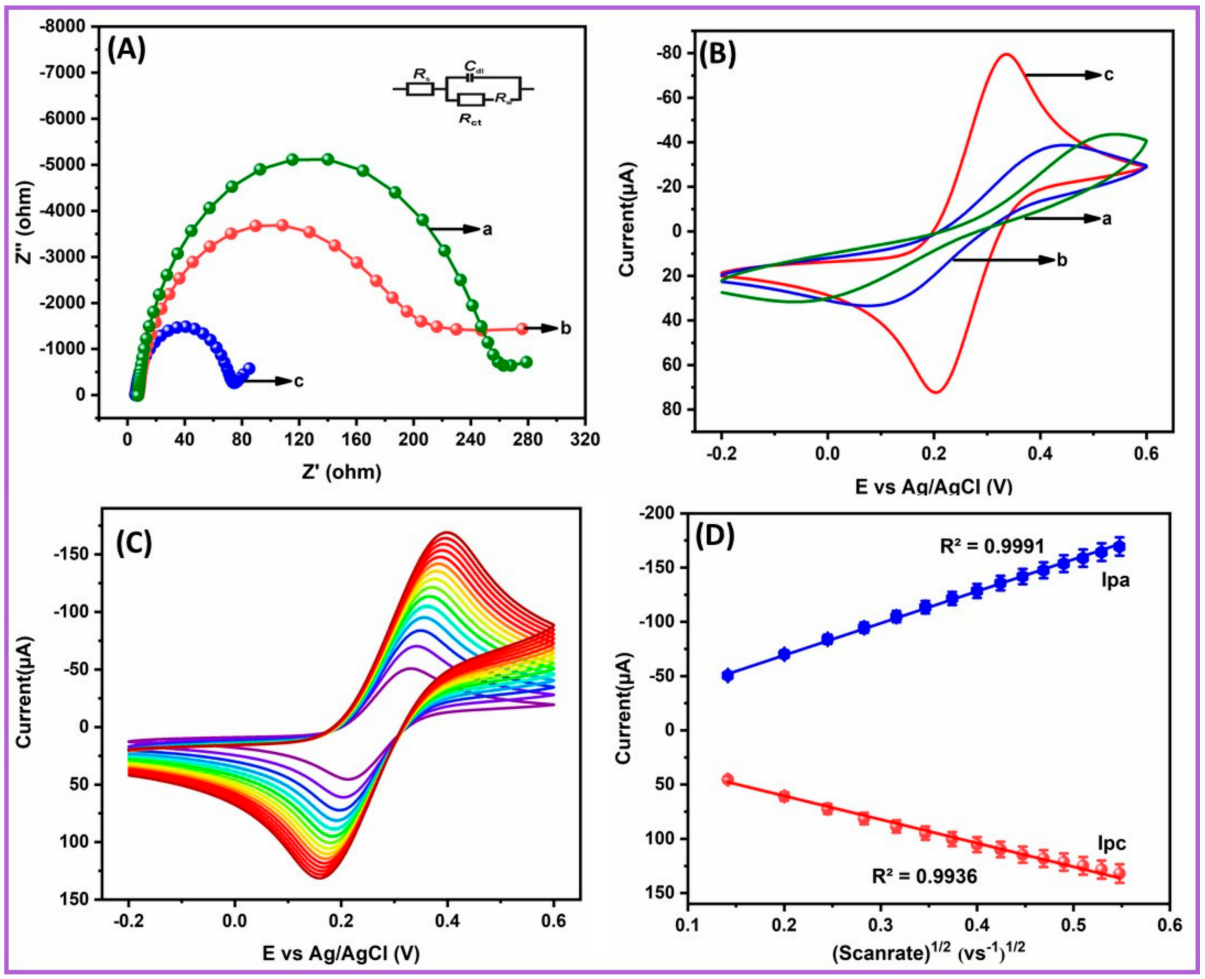
(**A**) EIS and (**B**) cyclic voltammetry (CV’s) (**a**) of unmodified electrode, (**b**) NiCo_2_O_4_-MFs/GCE and (c) NiCo_2_S_4_-Ms/GCE in 0.1-M KCl containing 0.05 M of [Fe(CN)_6_]^−3/−4^. (**C**) Scan rate of the nanocomposite modified electrode. (**D**) Calibration plot of (*v* vs. Vs^−1^)^1/2^.

Electrochemical impedance spectroscopy (EIS) is a method to investigate the interfacial effects between electrolyte and surface of the electrode. Figure 4A depicts the EIS curves of bare GCE (a), NiCo_2_O_4_-MFs/GCE (b) and NiCo_2_S_4_-Ms/GCE (c) in 0.1-M KCl containing 0.05 M of [Fe(CN)_6_]^−3/−4^ solution and the frequency range was set to be of 100 MHz to 100 kHz. The obtained impedance data were fit according to the Randle’s equivalent circuit model shows in the inset Figure 4A, where *R_ct_* denotes the charge transfer resistance, *Z*_W_, *R*_s_ and *C*_dl_ refer to Warburg impedance, ohmic resistance and the double layer electron-transfer resistance, respectively. Moreover, the *R_ct_* value of bare GCE, NiCo_2_O_4_-MFs/GCE and NiCo_2_S_4_-Ms/GCE were measured to be 250.7 Ω, 214.2 Ω and 64.25 Ω, respectively. As can be seen that the lowest *R_ct_* value were obtained for NiCo_2_S_4_-Ms electrode. This was due to the microsphere structure possessing the larger surface area; when it contacted the electrolyte, it may be more effective and efficient in capturing the active materials.

In addition, the cyclic voltammetry (CV’s) were performed to investigate the electrochemical behavior of bare GCE (a), NiCo_2_O_4_-MFs/GCE (b) and NiCo_2_S_4_-Ms/GCE (c) in 0.1-M KCl containing 0.05 M of [Fe(CN)_6_]^−3/−4^ solution. In Figure 4B as can be seen that, the highest reduction and reduction peak current and lower peak-to-peak separation (∆Ep) value of 89.24 mV were observed for the NiCo_2_S_4_-Ms/GCE. Moreover, the peak-to-peak separation (∆Ep) of unmodified electrode (a) and NiCo_2_O_4_-MFs/GCE (b) were measured to be 189.51 and 145.36 mV, respectively. Figure 4C shows the different scan rates at NiCo_2_S_4_-Ms/GCE, in which the redox peak current increased consistently and their linear relationship with the square root of scan rates were plotted as shown in Figure 4D. Furthermore, the electrochemical active surface area value was measured based on the Randel’s Sevcik equation (I). The electrochemical active surface area of bare GCE (a), NiCo_2_O_4_-MFs/GCE (b) and NiCo_2_S_4_-Ms/GCE were calculated to be 0.082, 0.102 and 0.161 cm^2,^ respectively. The results indicate the combination of bimetallic sulfides increased the surface area and showed excellent electrochemical contact between the surface of electrode and electrolyte solution.

Ip = 2.69 × 10^5^n^3/2^AD^1/2^C*v*^1/2^ (1)

### 2.4. Electrochemical Activity and Different pH at NiCo_2_S_4_-Ms/GCE

**Figure 5:**
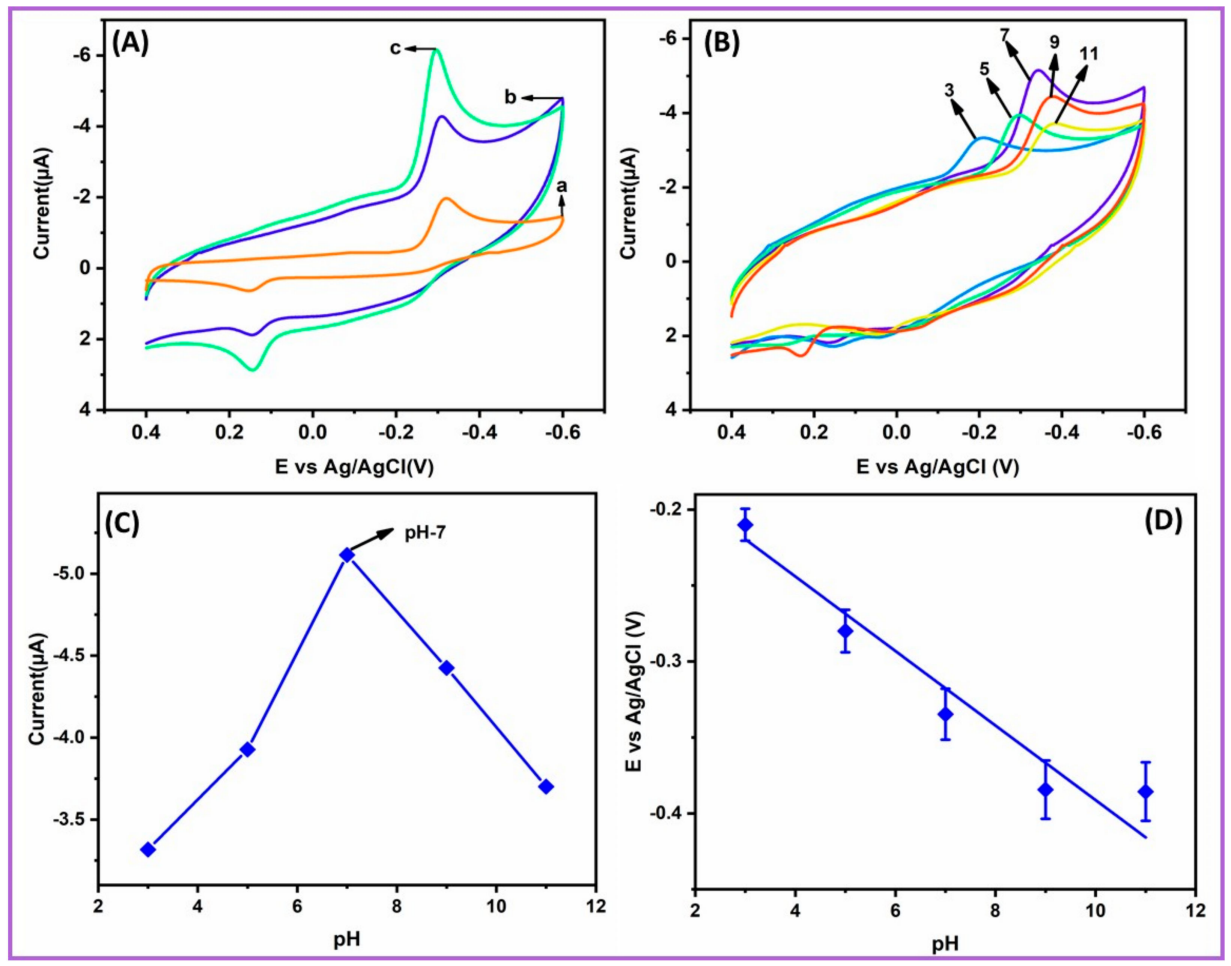
(**A**)—(**a**) CV’s of unmodified glass carbon electrode (GCE), (**b**) NiCo_2_O_4_-MFs/GCE, and (**c**) NiCo_2_S_4_-Ms/GCE in 0.1-M pH 7 containing 100-µM 4-NQO. (**B**) CV’s obtained at NiCo_2_S_4_-Ms/GCE for the varied pH from (3 to 11) containing 100-µM 4-NQO. (**C**) The plot current (µA) versus pH and (**D**) potential versus pH.

**Figure 6:**
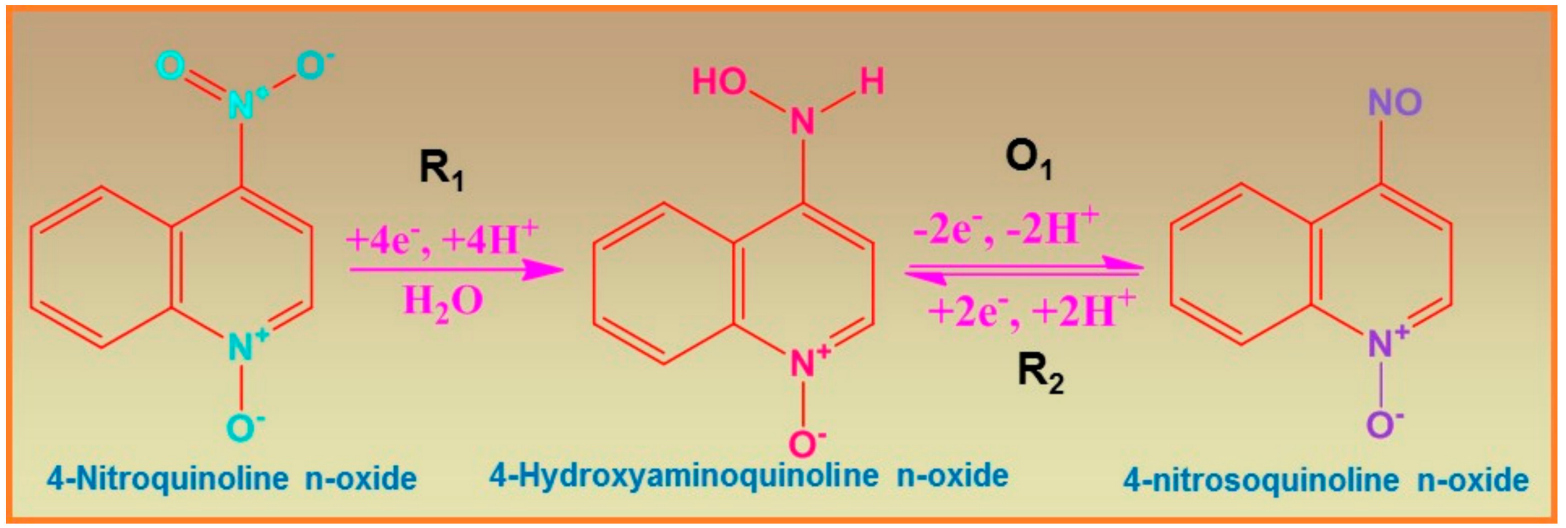
Possible reduction mechanism of 4-NQO at NiCo_2_S_4_-Ms/GCE.

As shown in Figure 5A, the CV’s with different electrodes, bare GCE (a), NiCo_2_O_4_-MFs/GCE (b) and NiCo_2_S_4_-Ms/GCE (c) were recorded in 0.1 M of phosphate buffer solution (PB) pH 7 containing 100-µM 4-NQO with the fixed scan rate of 0.05 V/s. The lower reduction peak current exhibited an unmodified GCE, which suggest the poor conductivity on the electrode surface. Furthermore, when the electrode was modified with NiCo_2_O_4_-MFs/GCE there was a drastically increase in the current. The excellent peak current of −26 µA with the minimized reduction potential of −0.29 V was noted on NiCo_2_S_4_-Ms/GCE, which suggest that the larger surface area of NiCo_2_S_4_-Ms can considerably enhance the sensitivity of the electrode. Moreover, the probable electrochemical reduction mechanism of 4-NQO on NiCo_2_S_4_-Ms/GCE are shown in Figure 6. Typically, 4-nitroquinoline n-oxide is irreversibly reduced to form 4-hydroxyaminoquinoline n-oxide. After this, the oxidation of 4-hydroxyaminoquinoline n-oxide occurs, which is subsequently reduced to 4-nitrosoquinoline n-oxide at the increasing scan. The accumulation time is one of the important parameters in electrochemical sensors to improve the sensitivity. Therefore, the accumulation time of the proposed sensor was studied in 0.1-M pH 7 containing 100-µM 4-NQO using the CV technique and the corresponding current response versus time were plotted as shown in Figure S2. The obtained results indicate that the reduction peak increased with the increasing accumulation time. However, the accumulation time exceeds 20 s, the reduction peak current of 4-NQO decreased. This was due to the adsorption taken at the surface of the electrode, which may have resisted the active site of the NiCo_2_S_4_-Ms and the surface reached its saturation point at 20 s. Thus, the optimal accumulation time of 20 s was preferred to achieve the high sensitivity for the proposed sensor.

Furthermore, the influence of pH at NiCo_2_S_4_-Ms/GCE for the detection of 4-NQO was investigated by varying the PB pH from 3 to 11 at a fixed scan rate of 0.05 Vs^−1^. Figure 5B displays the CV’s curves of different pH 3 to 11 containing 100-µM 4-NQO. The well-shaped peak with higher reduction peak current of −5.29 µA was noted at pH 7 Figure 5C. At some point, the peak current started to decrease, which indicates that the biomolecules reached its maximum pH value of 9–11. Hence, the pH 7 was chosen as the enhanced pH for the reduction of 4-NQO. In addition, the plot between the reduction peak potential and pH were plotted as shown in Figure 5D, which was linear, and its regression equation were represented as E_p_ (V) = −0.056 pH −0.624. The obtained slope value of −56 mV/pH indicates that the reaction was equal number of proton and electron transferred.

### 2.5. Influence of Different Concentration and Scan Rate

**Figure 7:**
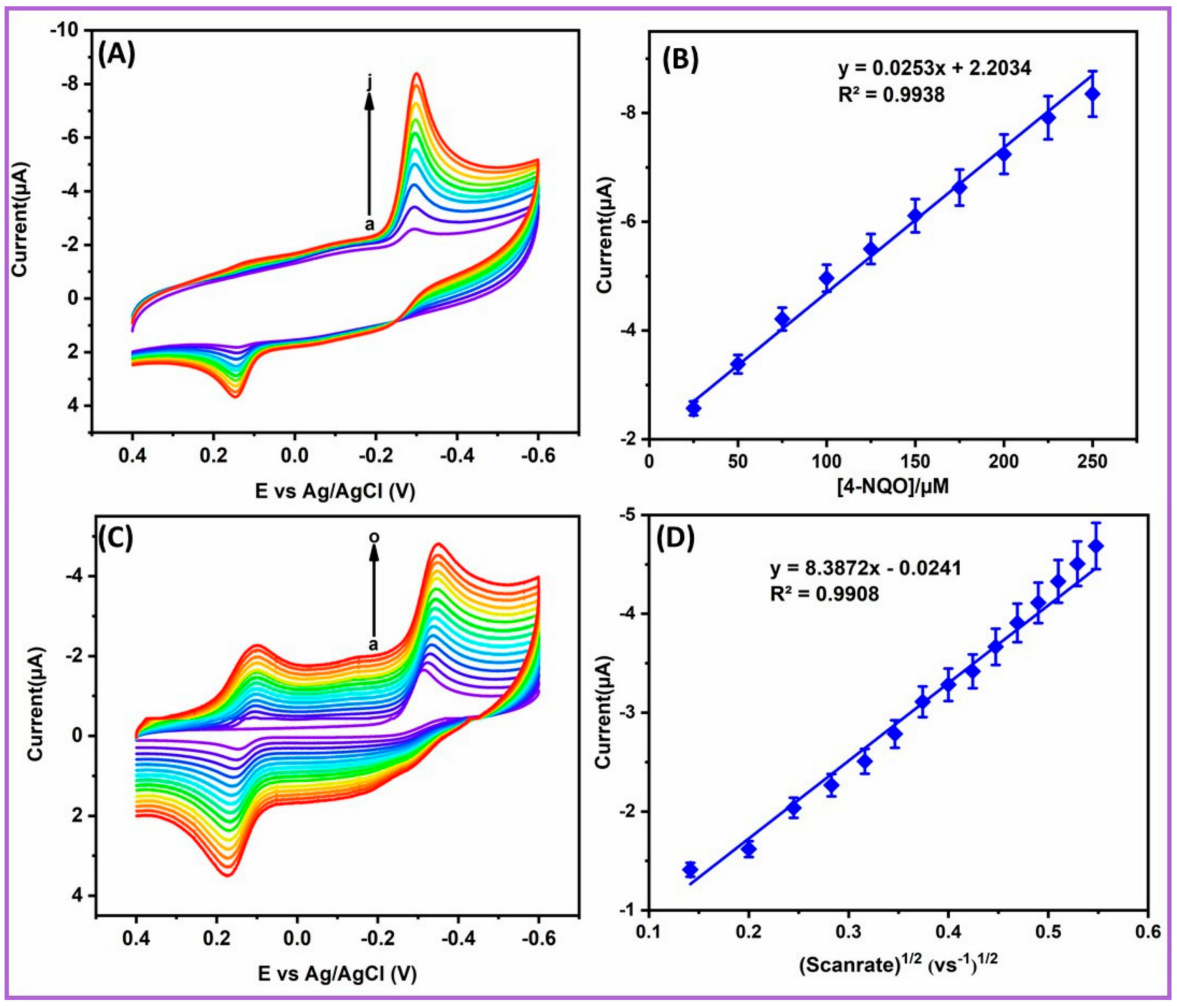
(**A**) CVs obtained at different concentration of 4-NQO (**a**–**j** 25–250 µM) and (**B**) Linear relationship between the current (µA) and 4-NQO concentration. (**C**) CVs attained for the different scan rate (**a**–**o** 20 to 300 mVs^−1^) in 0.1-M pH 7 at NiCo_2_S_4_-Ms/GCE. (**D**) Calibration plot of the cathodic peak current and scan rates.

In addition, the influence of various concentration of 4-NQO at as-prepared NiCo_2_S_4_-Ms/GCE were analyzed in 0.1-M pH 7 at fixed scan window of 0.05 Vs^−1^. As shown in Figure 7A, the CV curve were recorded for the increasing concentration of 4-NQO. For every sequential addition of 4-NQO from 25 to 250 µM the reduction peak current also increased linearly. Finally, the linear relationship of reduction peak current versus the concentration of 4-NQO were plotted as Figure 7B. Moreover, the regression equation is written to be *y* = 0.0253x + 2.2034 with the correlation coefficient of *R*^2^ = 0.9938. The obtained results confirm that, the as-prepared NiCo_2_S_4_-Ms is a promising electrode material for the rapid detection of 4-NQO without fouling.

In order to study the effects of scan rates at NiCo_2_S_4_-Ms/GCE was investigated by CV techniques. Figure 7C, indicates the CVs of different scant rates from 20 to 300 mVs^−1^ in 0.1-M PB pH 7 contains 100-µM 4-NQO.With consistently increasing scan rates, there was a linear increase in the cathodic peak current. Figure 7D shows the linear relationship between the cathodic peak current of 4-NQO versus square root of the scan rates. In addition, the regression equation was calculated to be *y* = 8.3872x − 0.0241 with the correlation coefficient of *R*^2^ = 0.9908, which indicates that reduction of 4-NQO at NiCo_2_S_4_-Ms/GCE was a diffusion-controlled process.

### 2.6. DPV Analysis of 4-NQO Ions at NiCo_2_S_4_-Ms/GCE Techniques

**Figure 8:**
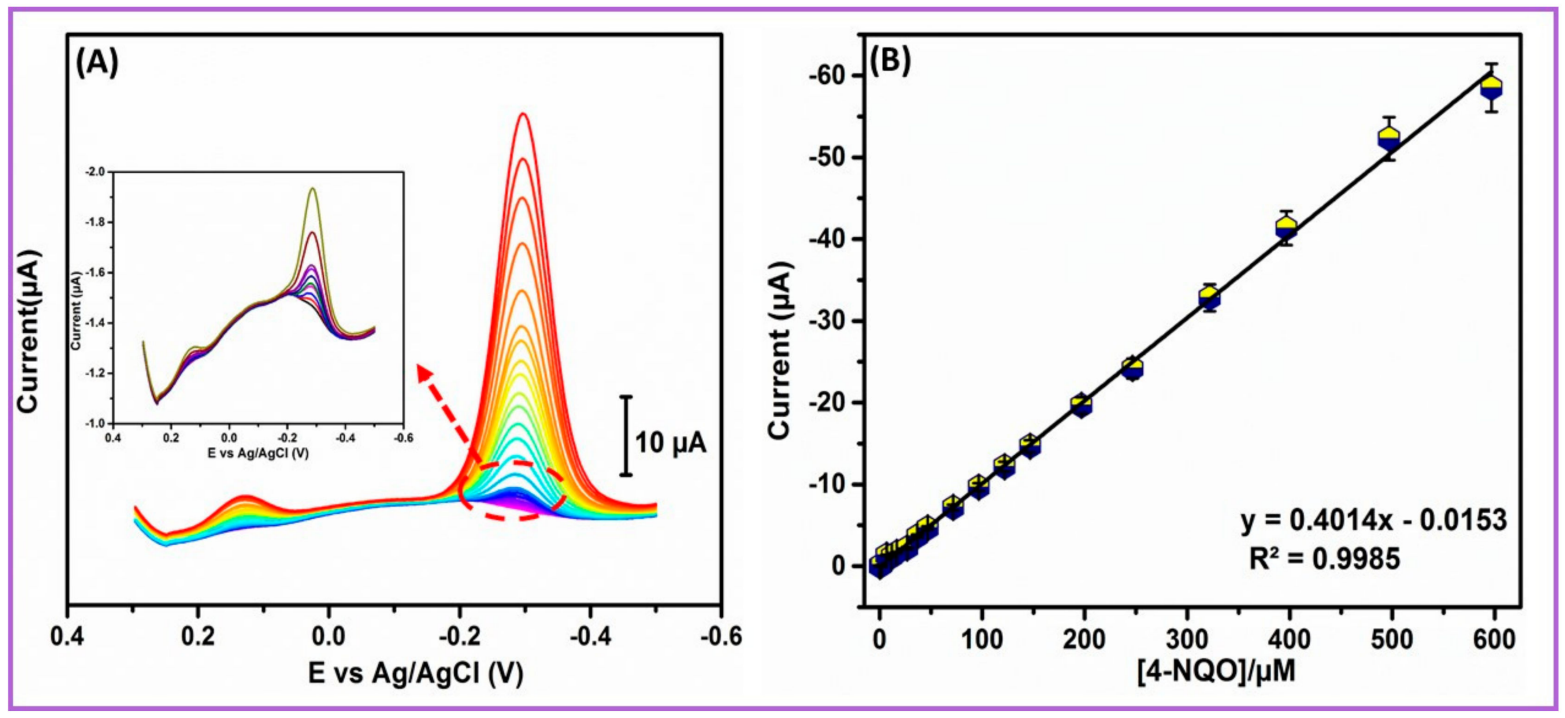
(**A**) DPV response of the different concentration of 4-NQO; (**B**) linear response of the reduction peak current versus concentration of 4-NQO.

Differential pulse voltammetry common techniques were used to measure the essential electrochemical parameters such as linear range, sensitivity and limit of detection (LOD). Hence, the DPV technique was used to detect the 4-NQO at NiCo_2_S_4_-Ms/GCE in 0.1-M pH 7 at a fixed potential of 0.4 to −0.6 V. Figure 8A displays the DPV curve of increasing concentration of 4-NQO. The obtained results indicate the reduction peak current increased with increasing concentration of 4-NQO. The NiCo_2_S_4_-Ms/GCE modified electrode showed an excellent linear relationship between the reduction peak current and the concentration of 4-NQO, which were plotted as shown in Figure 8B. Moreover, the linear regression equation was calculated to be *i*p_a_ (μA) = 0.4014 μM –0.0153 (*R*^2^ = 0.9985). Wherein, the NiCo_2_S_4_-Ms/GCE shows the wider range of 0.005 to 596.64-µM 4-NQO. In addition, the LOD of the as-prepared sensor was measured using the standard formula of LOD = 3σ/S, where the σ and S, is the standard deviation and slope of the curve, respectively. Moreover, the LOD of NiCo_2_S_4_-Ms/GCE for the detection of 4-NQO were measured to be 2.29 nM. Moreover, the proposed sensor wes compared with previously reported 4-NQO sensor as the results as-prepared NiCo_2_S_4_-Ms/GCE shows the very low detection limit Table 1. Thus, it is a promising electrode material for the detection of 4-NQO.

### 2.7. Interference and Stability Studies of NiCo_2_S_4_-Ms/GCE

**Figure 9:**
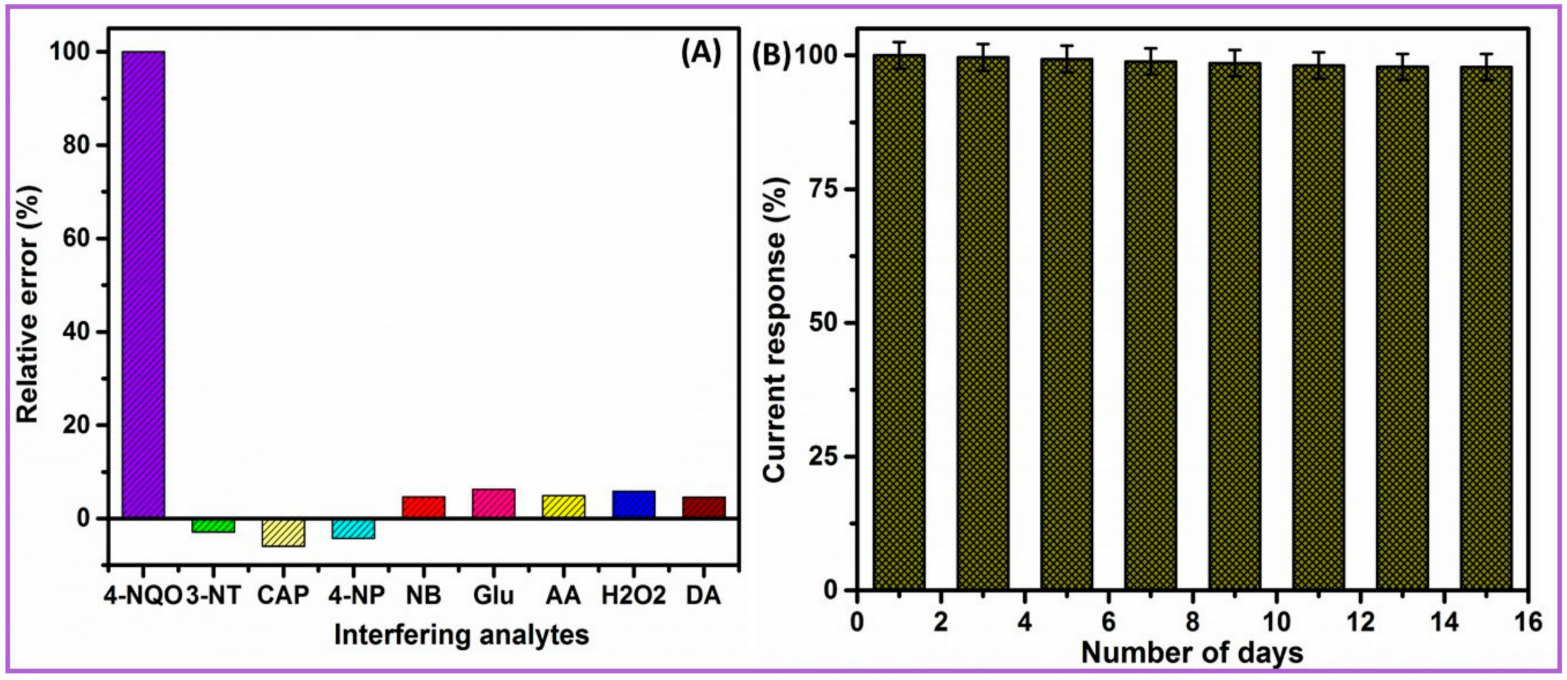
(**A**) Influence of interfering ions in 0.1-M pH 7 containing 0.5 µM of 4-NQO and higher concentration of interfering analytes; (**B**) stability plot of NiCo_2_S_4_-Ms/GCE of its usage for 15 days.

Selectivity and stability are the most considered parameter in electrochemical studies. Hence, the selectivity of the sensor was examined using NiCo_2_S_4_-Ms/GCE with 4-NQO, as well as in the presence of possible bioactive and nitro compounds. The selectivity of the prepared sensor was studied using DPV techniques. The DPV results of the NiCo_2_S_4_-Ms/GCE in presence of 0.5 µM 4-NQO and 0.7 µM possible interferents such as 3-nitro-l-tryosine (3-NT), 4-nitrophenol (4-NP), chloramphenicol (CAP), nitro benzene (NB) and continued addition of 1.5 µM common biologic analytes glucose (Glu), ascorbic acid (AA), hydrogen peroxide (H_2_O_2_) and dopamine (DA) are shown in Figure S3. The related nitro compounds such as 3-NT, 4-NP, NB, CAP showed minor interference with the 4-NQO due to structural similarity. However, the corresponding relative error was found less than 7% as shown in Figure 9A. Notably, most of the other nitro compounds show their reduction peaks at high overpotential of −0.50 to −0.60 V, which is far from the reduction potential of 4-NQO. In other words, although all these compounds have nitro groups, they require different energy to be reduced, which provides the electrode a good selectivity. Approximately three-fold excess concentrations of Glu, AA, H_2_O_2_ and DA did not show any significant interference, indicating that the method is selective in biologic samples. Moreover, the working stability of NiCo_2_S_4_-Ms/GCE were examined using the DPV technique. Figure 9B shows the stability current response of the prepared sensor in 0.1 M pH 7 containing 10-µM 4-NQO. After 15 days usage, the sensor maintained a stability of 94.59%. Thus, the stability test of the NiCo_2_S_4_-Ms/GCE confirms the outstanding working stability for the detection of 4-NQO.

### 2.8. Real Sample Analysis

The practical applicability of prepared sensor was examined in the biologic sample human blood serum and saliva samples. The preparation of biologic samples was diluted with buffer solution and the known concentration of 4-NQO were spiked. Finally, DPV techniques were carried out for the prepared real samples. Fascinatingly, the blood serum and saliva samples were showed outstanding found and recovery rates, which are tabulated in Table 2. At the end, the prepared NiCo_2_S_4_-Ms/GCE established as the effective electrode for the real time applicability.

## 3. Experimental Section

### 3.1. Synthesis Method for NiCo_2_S_4_-Ms and NiCo_2_O_4_-MFs

**Scheme 1:**
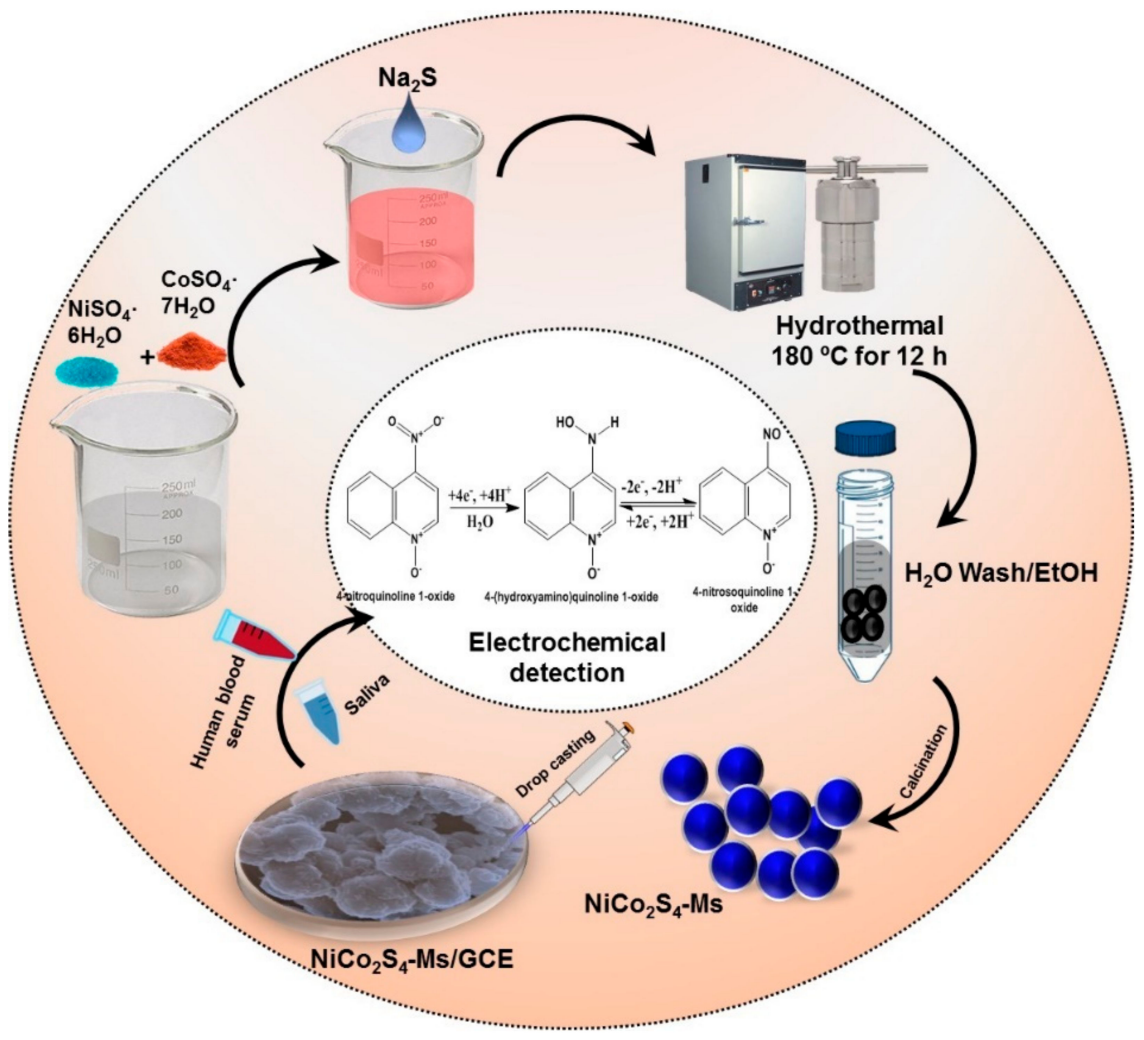
Synthesis methodology of NiCo_2_S_4_-Ms and its electrocatalytic properties for the detection of 4-nitroquinoline n-oxide.

All chemicals were purchased from Sigma-Aldrich in analytical grade and used without any further purification. The details of chemical purchases, preparation methods of buffer solutions and instrumentation techniques are detailed in the supplementary data (S1). The NiCo_2_O_4_-MFs and NiC_2_S_4_-Ms were synthesized by the hydrothermal method. Briefly, a 1:2 molar ratio of Ni(CH_3_COO)_2_·4H_2_O and Co(CH_3_COO)_2_·4H_2_O were thoroughly dissolved in 50 mL of C_2_H_6_O_2_ under magnetic stirring for 30 min. Then the mixture was carefully transferred into a 100 mL Teflon-lined stainless-steel autoclave and maintained for 180 °C for 12 h in a hydrothermal oven. After 12 h, the reaction mixture was allowed to cool room temperature and centrifuged with water and ethanol to collect the NiCo_2_O_4_-MFs precipitate. Finally, the collected precipitate was dried in air at 80 °C for 10 h [30]. To synthesis, the NiCo_2_S_4_-Ms, a 1:2 molar ratio of the NiSO_4_·6H_2_O and CoSO_4_·7H_2_O were dissolved in 0.5 M Na_2_S·5H_2_O. Then the same procedure was followed to obtain the NiCo_2_S_4_-Ms (Scheme 1).

### 3.2. Fabrication of NiCo_2_S_4_-Ms Modified GCE

The surface of GCE was cleaned by 0.5 mg of alum in a slurry. Then the GCE was dipped into an ethanol solution and sonicated for 10 min. After this, the GCE was pre-cleaned by cycling between −0.8 to 0.8 V in PB pH 7 for the 25 continuous cycle. Then, 1 mg of as-prepared NiCo_2_S_4_-Ms was dispersed in 1 mL of ethanol and sonicated for 20 min. Approximately 6 µL of NiCo_2_S_4_-Ms suspension was drop-cast on the surface of GCE and dried in a 50 °C oven. Finally, the as-prepared NiCo_2_S_4_-Ms modified GCE was used for the electrochemical characterization.

## 4. Materials and Reagents

Nickel acetate Ni(CH_3_COO)_2_·4H_2_O, cobalt acetate Co(CH_3_COO)_2_·4H_2_O, ammonium hydroxide (NH_4_OH), nickel sulfate NiSO_4_·6H_2_O, cobalt sulfate CoSO_4_·7H_2_O, 4-nitroquinoline n-oxide, uric acid (UA), ascorbic acid (AA), dopamine (DA), glucose (GLU), 3-nitrotyrosine (3-NT), L-cysteine and H_2_O_2_ and all other chemicals were purchased from Sigma-Aldrich and used as received. Double-distilled water was used for all the experiments. 0.1-M phosphate buffer (PB) (pH 7.0) prepared from sodium dihydrogen phosphate and disodium hydrogen phosphate was used as supporting electrolyte. Human blood serum and saliva samples were acquired from Chang Gung Medical Hospital, Taoyuan, Taiwan. The research protocols of human blood serum and saliva samples experiments were followed as per the laws and institutional guidelines of Chang Gung Medical Hospital (CGMH), Taiwan.

## 5. Methods

The surface modification of the as-formed composite was examined using field emission scanning electron microscope (FESEM-JEOL-7600F, Jeol instruments, Musashino, Akishima, Tokyo, Japan): Ingredients of the elemental composition and elemental mapping were analyzed by energy-dispersive X-ray spectroscopy (EDX) with HORIBA EMAX X-ACT (Horiba instruments, AkzoNobel House, Singapore). The quantitative analysis and defects and disordered nature of the as-prepared composite were investigated by PerkinElmer PHI-5702 (PerkinElmer Inc., Waltham, MA, USA).

The crystalline nature of the composite was examined by XRD, XPERT-PRO spectrometer (Malvern Panalytical B.V., Almelose Aa, Netherland). The electrochemical properties and electrocatalytic activity were examined using electrochemical impedance spectroscopy (EIS) and cyclic voltammetry (CV); i-t amperometry was carried out CHI 1205A (CH instruments, Inc. Austin, TX, USA). The CHI instrument consists of a three-electrode system, in which the platinum wire and Ag/AgCl (sat. KCl) were used as auxiliary and reference electrodes, and a pre-washed GCE (glassy carbon electrode) acted as a working electrode.

## 6. Conclusions

In summary, we successfully synthesized NiCo_2_S_4_-Ms and NiCo_2_O_4_-MFs through the hydrothermal method. The formation of NiCo_2_S_4_-Ms and NiCo_2_O_4_-MFs were conformed through the several analytical techniques such as FESEM, EDX, XPS and XRD. Further, the as-prepared nanocomposites were used as electrode materials for the detection of 4-NQO. Fascinatingly, the NiCo_2_S_4_-Ms modified electrode showed excellent electrochemical performances such as a wider range, low limit detection, higher selectivity and outstanding stability for the detection of 4-NQO. In addition, the practical applicability of the as-prepared nanocomposite were scrutinized in the human blood serum and saliva samples, which showed good recovery for both samples. To our knowledge, the hydrothermally synthesized NiCo_2_S_4_-Ms is the one of the most effective electrocatalysts for the detection of 4-NQO.

## Figures and Tables

**Table 1:** Comparison of as-prepared sensor with the other previously reported 4-NQO sensor.

Method	Linear Range (µM)	LOD (nM)	Ref
Bi_2_WO_6_/rGOs·NC/^a^ GCE/^b^ it	0.025–718	6.11	[22]
Fe_2_ N·NPs@rGOS/^c^ SPCE/^d^ DPV	0.05–574.2	9.24	[16]
ZnFe_2_O_4_·NCs/rGO/^a^ GCE/^d^ DPV	0.025–534.12	8.27	[19]
^e^ HPLC	-	0.157	[29]
NiCo_2_S_4_-Ms/GCE/DPV	0.005–596.64	2.29	This work

^a^ Glassy carbon electrode; ^b^ (i–t) amperometric; ^c^ screen printed carbon electrode; ^d^ differential pulse voltammetry; ^e^ high-performance liquid chromatography.

**Table 2:** Determination of 4-NQO in biologic samples at NiCo_2_S_4_-Ms/GCE modified electrode.

Real Samples	Added/nM	Found/nM	Recovery/%	* RSD/%
Human blood serum	50	47.58	95.16	2.45
	100	96.48	96.48	2.81
Saliva	50	49.12	98.24	2.56
	100	94.29	94.29	2.76

* Related standard deviation (RSD) of *n* = 3.

## Supplementary Materials

The following are available online at https://www.mdpi.com/1422-0067/21/9/3273/s1, Figure S1. Mapping image of NiCo2O4-MFs (A). (B-D) conforms the presence of Ni (B), Co (C), O (D). (E) EDX profile for NiCo2O4-MFs; Figure S2. Effect of accumulation time on the reduction peak current of 100 µM 4-NQO at NiCo2S4-MS/GCE; Figure S3. DPV curve of obtained at NiCo2S4-Ms/GCE in the 0.1M pH-7 containing 0.5 µM of 4-NQO with other bioactive and electro active nitro species.
