# Supplementary material for: An Ultra-Sensitive Electrochemical Sensor for the Detection of Carcinogen Oxidative Stress 4-Nitroquinoline N-Oxide in Biologic Matrices Based on Hierarchical Spinel Structured NiCo2O4 and NiCo2S4; A Comparative Study
Source: Int J Mol Sci. 2020 May 5;21(9):3273. doi: 10.3390/ijms21093273 (PMC7247362; doi:10.3390/ijms21093273)
Supplement: Supplementary file 1 [file ijms-21-03273-s001.pdf]

Supporting information

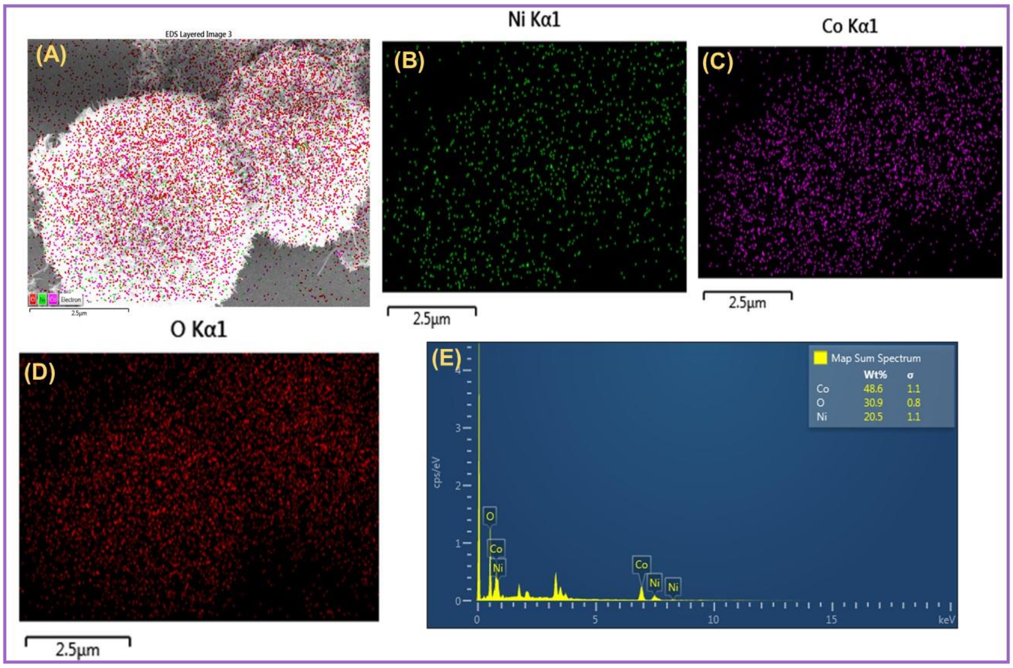

Figure S1. Mapping image of $NiCo_2O_4$-MFs (A). (B-D) conforms the presence of Ni (B), Co (C), O (D). (E) EDX profile for $NiCo_2O_4$-MFs. Inset: weight perctange.

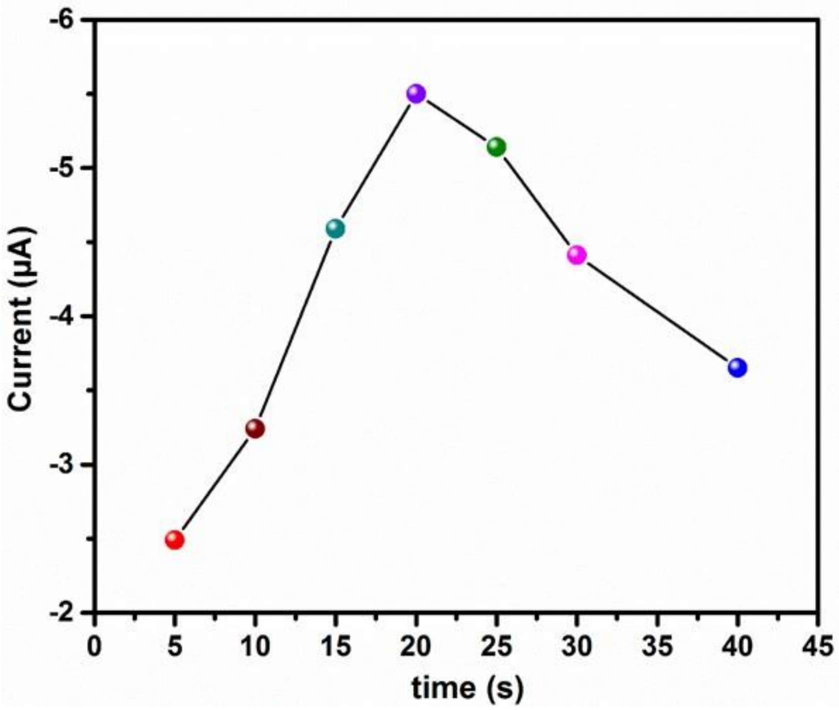

Figure S2. Effect of accumulation time on the reduction peak current of 100 μM 4-NQO at $NiCo_2S_4$-MS/GCE.

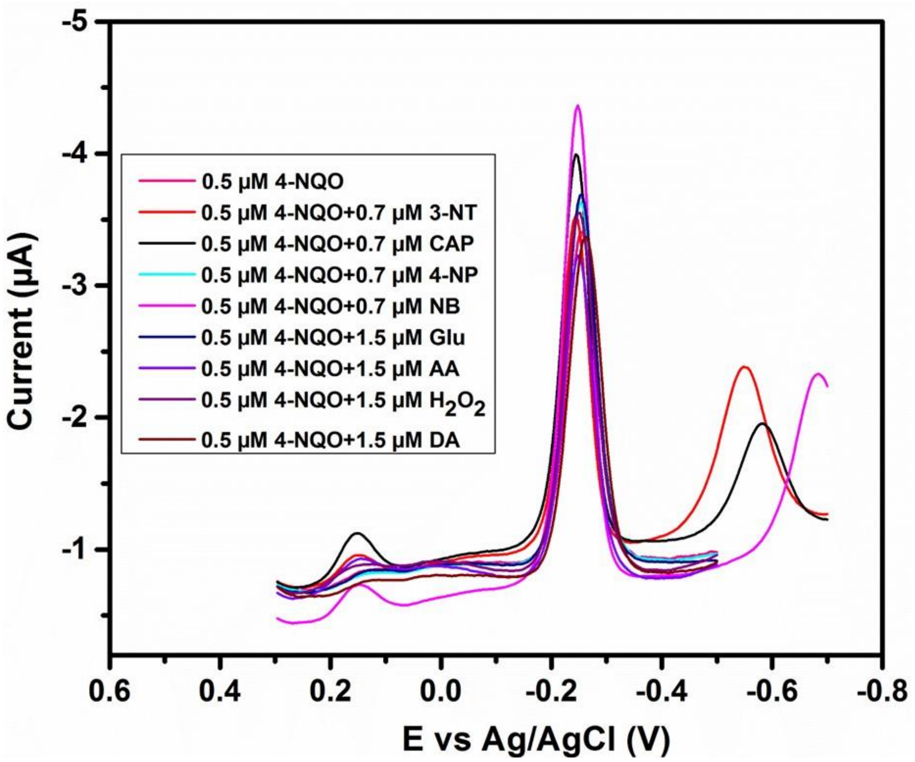

Figure S3. DPV curve of obtained at $NiCo_2S_4$-Ms/GCE in the 0.1M pH-7 containing 0.5 μM of 4-NQO with other bioactive and electro active nitro species.
